# OTFS Radar Waveform Design Based on Information Theory

**DOI:** 10.3390/e27020211

**Published:** 2025-02-17

**Authors:** Qilong Miao, Ling Kuang, Ge Zhang, Yu Shao

**Affiliations:** School of Information and Communication Engineering, University of Electronic Science and Technology of China, Chengdu 611731, China; miaoqilong20240021@outlook.com (L.K.); zhangge12211201@outlook.com (G.Z.); yss202222010608@outlook.com (Y.S.)

**Keywords:** radar, OTFS, waveform design, information theory, conditional mutual information

## Abstract

In this work, we consider the waveform design for radar systems based on orthogonal time–frequency space (OTFS). The conditional mutual information (CMI), chosen as a promising metric for assessing the radar cognitive capability, serves as the criterion for OTFS waveform design. After formulating the OTFS waveform design problem based on maximizing CMI, we propose an equivalent waveform processing approach by minimizing the autocorrelation sidelobes and cross-correlations (ASaCC) of the OTFS transmitting matrix. Simulation results demonstrate that superior performance in target information extraction is achieved by the optimized OTFS waveforms compared to random waveforms.

## 1. Introduction

Orthogonal time–frequency space (OTFS), a two-dimensional (2D) modulation scheme that exhibits superior performance compared to orthogonal frequency-division multiplexing (OFDM) in high-mobility channel environments, is a promising solution for rapidly changing communications [1,2] and radar applications [3,4,5]. Currently, there exist several methods for OTFS waveform design [6,7]. Recently, it was verified that OTFS cannot exploit the full delay and Doppler diversity. However, to the best of our knowledge, there is currently no OTFS waveform design method that adopts CMI as a criterion. The reason for OTC-based radar applications is that OTFS has a higher tolerance to Doppler shifts; thus, it could mitigate Doppler effects and enhance target detection and tracking accuracy. Also, it has a higher delay-Doppler resolution, thus resolving targets in both range and velocity directions accurately even in multi-target scenarios. Moreover, OTFS has great potential for waveform designs, optimizing them for specific radar applications and/or enhancing their performance and efficiency, as well as allowing use for integrated communication-radar systems, in which systems can share the waveform, reducing the complexity and cost and improving the performance. This makes OTFS a suitable candidate for radar applications due to its promising capability to develop radar systems that can work well in many scenarios through leveraging the benefits of OTFS with better performance than legacy modulation designs and the modulation schemes used for communication systems.

Information theory stands as a vital tool for radar waveform design [8,9]. The roots of radar waveform design based on information theory can be traced back to [8]. In their works, conditional mutual information (CMI) between the random target response and the received signal is adopted as a criterion. Building upon this foundation, ref. [9] expanded the application of information theory-based waveform design to multiple-input multiple-output (MIMO) radar systems for extended targets. The main reason for choosing information theory in radar waveform design is its capacity to evaluate a radar’s information acquisition ability, which directly correlates with improved target recognition capabilities.

The CMI was used in this work because of its capacity to quantify how much information could be obtained regarding the target by using a radar system, given prior knowledge of the target’s reflectivity and noise. Fundamentally, depending on the signal, CMI can be used to design a radar wave-form that most effectively extracts the information of a target that helps to detect, recognize, and track the target. CMI has several advantages for the analysis in this work: this measure can provide a fixed lower limit on the performance of the radar system that is independent of the waveform design; it can also be extended to represent a wide variety of target and noise models, making it an effective measure to inform and optimize radar waveform design. Additionally, CMI offers a systematic approach to assess the capabilities and limitations of various radar waveforms, allowing for direct comparison and resulting in the design of superior radar systems.

The design of the OTFS waveform would need to contemplate communication complexity in addition to MM and NN dimensional optimization. With larger matrices, more variables have to be optimized, making optimization exponentially harder. On the other hand, larger dimensions give more freedom in optimizing the waveform, allowing potentially better performance. Techniques, including parallel processing and distributed optimization, could be used to efficiently scale the design. Additionally, dimensionality reduction techniques could be utilized to narrow down the number of variables to optimize over, making the problem more manageable. The OTFS waveform can then be designed by simply up-scaling concerning the extended dimensions of the OTFS transmitting matrix, thus leading to advanced and high-performance radar systems.

The novelties in our work are as follows:

- We derived the CMI between received OTFS signals and Gaussian extended targets and formulated the OTFS waveform design problem based on maximizing CMI under constant modulus constraint conditions. To the best of our knowledge, this is the first instance in which CMI has been applied to the OTFS waveform design problem.
- Based on the characteristics of the OTFS transmitting shift matrix, we formulated the waveform design problem aiming to minimize auto-correlation sidelobes and cross-correlations (ASaCC). This problem can be efficiently solved by Multiple Cyclic Algorithm-New (Multi-CAN) [10].

The remaining part of this paper is organized as follows. In Section 2 and Section 3, the problem of OTFS waveform design is formulated and solved. The simulation and conclusions are presented in Section 4 and Section 5, respectively.

## 2. Problem Formulation

### 2.1. OTFS-Based Radar Model

Extended targets, as opposed to point targets, have echoes that carry structural information about the target. Consequently, the echoes from extended targets not only provide information about the presence of a target but also about the identity of the target itself. Therefore, in this work, our focus is specifically on extended targets. Extended targets are particularly important in various radar applications, including the following:

Airborne radar: While airborne radar systems typically illuminate extended targets, such as aircraft, ships, or terrain features, the large target cross-section calls for the radar system to resolve and track multiple scattering centers.

Ground-penetrating radar: GPR systems often have to deal with long-range targets, such as buried objects, tunnels, and underground structures, which places specific demands on the radar system to resolve and image intricate subsurface features [11].

SAR (synthetic aperture radar): SAR systems are typically used to image wide-area targets like buildings, roads, and vegetation, and these targets require the radar system to resolve and reconstruct complicated scenes. For an extended target containing P scatters, the noise-free echo is

(1) $$r\left(t\right)=\sum_{p=1}^{p}\alpha_{p}\exp\left(j2\pi f_{d_{p}}\right)x\left(t-\tau_{p}\right)$$

where x(t) is the OTFS-modulated transmitting signal, $\alpha_{p}$ is the scattering coefficient, $f_{d_{i}}$ is the Doppler, and $\tau_{i}$ is the delay. Unlike communication, the delays here correspond to a round-trip between the sensor and the target. Thus, the delay-Doppler response of the target is

(2) $$\rho \left(\tau ,f_{d}\right)=\sum_{p=1}^{p}\alpha_{p}\delta (T-T_{p})\delta (f_{d}-f_{d_{p}}).$$

**Figure 1:**
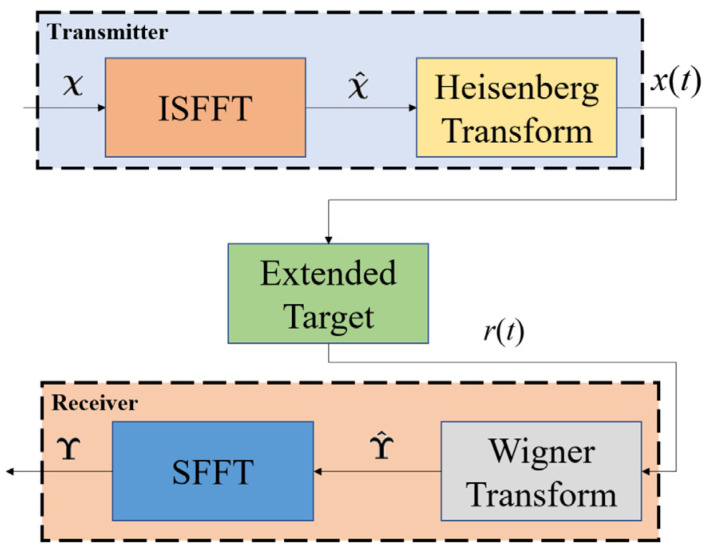
Block diagram of OTFS-based radar.

The diagram of the OTFS-based radar is depicted in Figure 1. Firstly, a delay-Doppler domain transmitting matrix $\chi \in C^{M\times N}$ is transformed into a time–frequency domain-transmitting matrix $\hat{\chi }\in C^{M\times N}$ using the inverse symplectic finite Fourier transform (ISFFT). Next, the Heisenberg transform is applied to $\hat{\chi }$ to generate x(t).

Following reflection by the extended target, the received signal r(t) is transformed into the time–frequency domain received matrix $\hat{ϒ}\in C^{\hat{N}\times \hat{M}}$ through the Wigner transform, where $\hat{M}$ and $\hat{N}$ represent the observation range. Finally, $\hat{ϒ}$ is converted into the delay-Doppler received matrix $ϒ\in C^{\hat{M}\times \hat{N}}$ using the symplectic finite Fourier transform (SFFT). For details regarding SFFT/ISFFT, the Heisenberg transform, and the Wigner transform, please refer to [2].

Under the assumptions of (A1) ideal transmitting and receiving pulses and (A2) the absence of fractional Doppler, following [2], the relationship between ϒ and χ is as follows:

(3) $$ϒ\left[m,n\right]=\sum_{p=1}^{p}\alpha_{p}\chi [[m-m_{i}{]}_{M,}\left[n-n_{i}{]}_{N}\right],$$

where $m_{i}$ is the index corresponding to the $\tau_{i}$ where $\tau_{i}=m_{i}\Delta t$, $n_{i}$ is the index corresponding to the Doppler $f_{d_{i}}$ with $f_{d_{i}}=n_{i}/NT$. Δt is the sampling interval, T is OTFS block duration, and $[•{]}_{M}$ is the modulo operation with M. By appropriately selecting $\hat{M}$ and $\hat{N}$, we can eliminate the need for the modulo operation. After eliminating the modulo operation and introducing noise, (3) becomes

(4) $$ϒ\left[m,n\right]=\sum_{p=1}^{p}\alpha_{p}\chi [\left[m-m_{i},n-n_{i}\right]+N\left[m,n\right]].$$

For a detailed elaboration on assumptions (A1) and (A2) and the derivation of (3), please refer to [2].

### 2.2. The CMI Between ϒ and the Extended Target

Let us consider the MI between ϒ and $\alpha =[\alpha_{1},\ldots ,\alpha_{p}{]}^{T}$, conditioned on χ. In this work, we operate under the following assumptions: (B1) α is a Gaussian random vector with zeros mean and diagonal covariance matrix $\Sigma_{\alpha }$. (B2) The noise N is additive white Gaussian noise with variance $\sigma_{0}^{2}$.

By defining χ[m,n]=0 for m<0 or n<0 and organizing χ as $\chi =[x_{1},\ldots ,x_{N}]$, (4) can be expressed as follows:

(5) $$ϒ=\sum_{p=1}^{p}\alpha_{p}\varrho_{p}\left(x\right)+N,$$

where $Q_{p}(\chi )\in C^{\hat{M}\times \hat{N}}$ relating to χ is

(6) $$Q_{p}\left(x\right)=\left[\begin{array}{ccc} 0_{M\times n_{i}} & \begin{array}{c} 0_{m_{i\times \hat{N}}}\\ \chi \\ 0_{\hat{M}-M-m_{i\times \hat{N}}} \end{array} & 0_{M\times \hat{N}-n_{i}-N} \end{array}\right]$$

Vectorize $Q_{p}(\chi )$ to $q_{p}(\chi )$ as

(7) $$q_{p}\left(x\right)=\begin{array}{cc} {}[0_{1\times n_{i}\hat{M}+m_{i}} & x_{1} \end{array}\;\;\;\;\begin{array}{cc} 0_{1\times \hat{M}-M} & x_{2}\;\;\;\ldots \end{array}{]}^{T}$$

Vectorize ϒ to y, where the element index $\hat{l}$ of y is $\hat{l}=m+n\hat{M}$. The noise vector n is obtained using the same approach.

From the OTFS transmitting shift matrix, $Q(\chi )=[q_{1}(\chi ),\ldots ,q_{p}(\chi )]$, (5) can be expressed as

(8) $$y=\varrho \left(x\right)\alpha +n.$$

Conditioned on χ, ref. [9] gives the conclusion of the CMI I(y,α|χ) in the form of (6) as follows:

(9) $$I\left(y;\alpha |X\right)=log[det(\sigma_{n}^{-2}\sum_{\alpha }Q\left(x{)}^{H}Q\left(x\right)+I_{\hat{N}}\right)].$$

Our goal is to design χ under a constant modulus condition to maximize I(y,α|χ). Therefore, the waveform problem can be expressed as

(10) $${max}_{x\;\;\;\;\;\;\;\;\;\;\;\;\;}\log\left[\det\left(\sigma_{n}^{-2}\sum_{\alpha }\varrho (x{)}^{H}\varrho (x)+I_{\hat{N}}\right)\right]\;s.t.\;\;x[i,j]*x\left[i,j\right]=1,i\;\epsilon \;\left\{1,\ldots ,M\right\},\;j\;\epsilon \;\{1,\ldots \},$$

### 2.3. OTFS Transmitting Matrix Design Based on CMI

Lemma 1 in [9] points out that under the assumptions of (B1) and (B2), the semi-positive definite hermit matrix $Q(\chi {)}^{H}$ is a diagonal matrix that will result in the maximization of I(y,α|χ). Therefore, our goal is to design χ in a manner that renders $Q(\chi {)}^{H}$ as a diagonal matrix.

Considering $\chi =[x_{1},\ldots ,x_{N}]$, $x_{i}=x_{i}(u)\in C^{M\times 1}$, we define the cross-correlation coefficients:

(11) $$Y_{i,j}(z)=\sum_{u=0}^{m}x_{i}^{*}\left(u\right)x_{j}\left(u-z\right)=Y_{ij}^{*}\left(-z\right).$$

Note that the elements $Q_{ij}$ of $Q(\chi {)}^{H}$ are related to the cross-correlation coefficients. Here, we focus on off-diagonal $Q_{ij}$. Recalling $Q_{p}(\chi )$, for different scatters i and j, there are three possible values of $Q_{ij}$: (1) If the delay and Doppler parameters cause the non-zero elements in both $Q_{p}(\chi )$ and $Q_{j}(\chi )$ to not overlap, then $Q_{ij}=0$, indicating that $Q(\chi {)}^{H}$ is diagonal. (2) If the Doppler of scatters i and j is the same while their delays differ,

(12) $$Q_{ij}=\sum_{n=1}^{N}\gamma_{n,n}\left(z\right).$$

where z≠0 is related to the Doppler difference of these two scatters. (3) If the Doppler and delays are not the same,

(13) $$Q_{ij}=\sum_{n=1}^{N}\gamma_{n,n+\kappa }\left(z\right).$$

where κ≠0 is related to delay difference and z is related to Doppler difference of these two scatters. Recall that our goal is to minimize off-diagonal elements $Q_{ij}$ in all possible cases, which is equivalent to minimizing both (10) and (11). To accomplish this, we employ the following optimization variable:

(14) $$\zeta =\sum_{n=1\;\;\;}^{N}\sum_{z=-M+1,z\neq 0}^{M-1}|\gamma_{n,n}(z){|}^{2}+\sum_{n_{1}=1}^{N}\sum_{n_{2}=1,\;n_{2}\neq n1}^{N}\sum_{z=-M+1}^{M-1}|\gamma_{n_{1,n2}}\left(z\right){|}^{2}.$$

Then, (8) is transformed into an equivalent optimization problem as

(15) $${min}_{x\;\;\;\;\;\;\;\;\;\;\;\;\;\;C}\;s.t.\;\;\;\;\;\;\chi [i,j]*\chi \left[i,j\right]=1,\;i\epsilon \left\{1,\ldots ,M\right\},j\epsilon \{1,\ldots ,N\}$$

(13) indicates that χ is designed to minimize ASaCC. Solving (13) can be achieved using Multi-CAN.

### 2.4. The Multi-CAN Algorithm

This work proposes the Multiple Cyclic Algorithm-New (Multi-CAN), which is a brand-new optimization algorithm to solve the formulated problem of the waveform design. The algorithm is an advanced version of the conventional Cyclic Algorithm-New (CAN) algorithm extensively used to solve optimization problems in diverse domains. In this part, a comprehensive description of the Multi-CAN algorithm and its use in the OTFS waveform design problem is presented.

#### 2.4.1. Multi-CAN Algorithm Overview

The Multi-CAN algorithm is employed by iteratively searching for an optimal solution and utilizing a combination of cyclic and gradient-based search techniques. The overall algorithm consists of two main parts, the cyclic search and a handful of gradient-based steps.

Auto-encoded Cyclic Search: The auto-encoded cyclic search uses cyclic search input to capture the relationships between different input features and update the optimization variables accordingly. The algorithm proceeds in iterations, updating each variable in the set individually while keeping the rest of the variables constant. This repeats until all variables have been updated.

Gradient-Based Search: This is a local optimization technique that uses the gradient of the objective function to search for the optimal solution. It allows the optimizing variables to update in the direction in which the objective function (used in the loss function) decreases using gradient information.

#### 2.4.2. Multi-CAN-Based OTFS Waveform Design Algorithm

To apply the Multi-CAN algorithm to the OTFS waveform design problem, we first express the optimization problem as follows:

ASaCC Metric: The ASaCC metric is responsible for satisfying the objective function, which is defined by the sum of autocorrelation sidelobes and cross-correlations of the OTFS transmitting matrix.

Optimization variables: The optimization variables correspond to the elements of the OTFS transmitting matrix.

The constraints are the constant modulus constraint and the constraints on the unitary OTFS transmitting matrix.

The ASaCC value can be minimized by organizing the iterative process of updating the elements of the OTFS transmitting matrix following the Multi-CAN algorithm. The algorithm changes one element for each iteration while keeping other elements constant. The update is performed with a mix of cyclic and gradient-based search approaches.

#### 2.4.3. Multi-CAN Algorithm Implementation

The steps for implementing the Multi-CAN algorithm to design the OTFS waveform are given as follows:

Setup: Generate a random or predetermined OTFS transmitting matrix.

Cyclic Search: Also known as a cycling search, it iterates through elements of the OTFS transmitting matrix, updating one element of the cycling search while fixing the others.

Search for OTSF Transmitting Matrices using an ASaCC Metric: Use an ASaCC metric and its gradient, gradient-based search.

OTFS transmitting matrix updating: Using the updated elements, update the OTFS transmitting matrix.

Repeat: Until convergence or a stopping criterion is met, repeat steps 2 to 4.

This also implies that OTFS induces a specific structure on the waveform, and it must therefore potentially be optimized in the delay-Doppler domain; this aspect also increases design complexity for OTFS while allowing us to increase flexibility and robustness to interference. This affects the waveform design by inferring a particular structure, used to optimize features like amplitude and phase in the delay-Doppler domain and obtain metrics in the operation. Therefore, we can determine the proper methods to apply OTFS to the waveform design, which will allow more effective and efficient development of radar systems that can work under a vast variety of situations.

## 3. Simulation and Analysis

In this section, we utilize the Multi-CAN to design χ, and subsequently compute the CMI for the given $\Sigma_{\alpha }$ and $\sigma_{0}^{2}$. Each initial $x_{i}$ is a random sequence with phases uniformly distributes in [0, 2π], and the modulus is 1.

Carrier frequency of 10 GHz and bandwidth of 100 MHz. The pulse duration is 10 μs, with a pulse repetition frequency of 100 kHz. Number of pulses = 100, OTFS frame duration = 1 ms, Size of OTFS frame = 1000 symbols, Modulation order = 4-QAM. The coding rate is 1/2. For the LFM waveform, chirp rate = 10^6^ Hz/Barker code (code length = 13) phase-coded waveform. It should be noted that the PAPR of the optimum waveform is a minimum of 2 dB, and the random waveform is uniformly distributed. With these waveforms, the performance of the OTFS waveform is compared against other existing waveforms.

**Figure 2:**
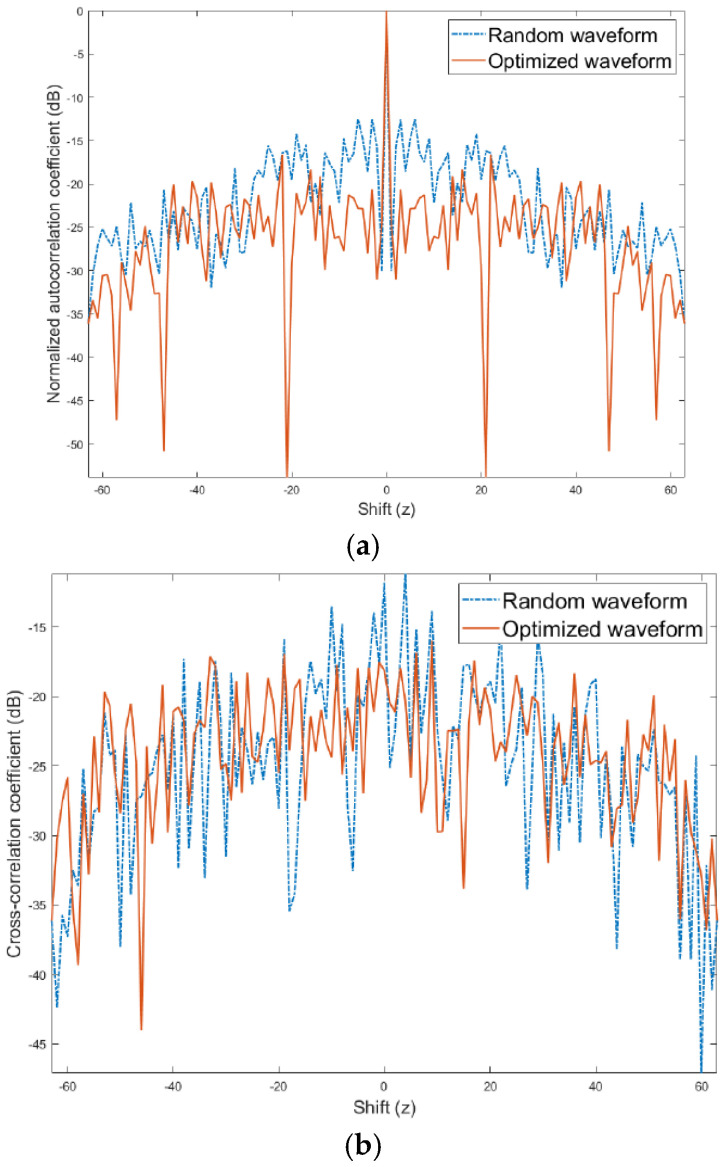
Multi-CAN results for M=64 and N=5 of (**a**) autocorrelation coefficients, and (**b**) cross-correlation coefficients normalized by the maximum value of the autocorrelation coefficients.

Figure 2 depicts the correlation coefficients of χ designed by Multi-CAN. Optimized χ exhibits lower ASaCC when compared to the initial random input. Multi-CAN yields approximately a 10 dB improvement in auto-correlation sidelobes and about a 5 dB improvement in cross-correlations.

**Figure 3:**
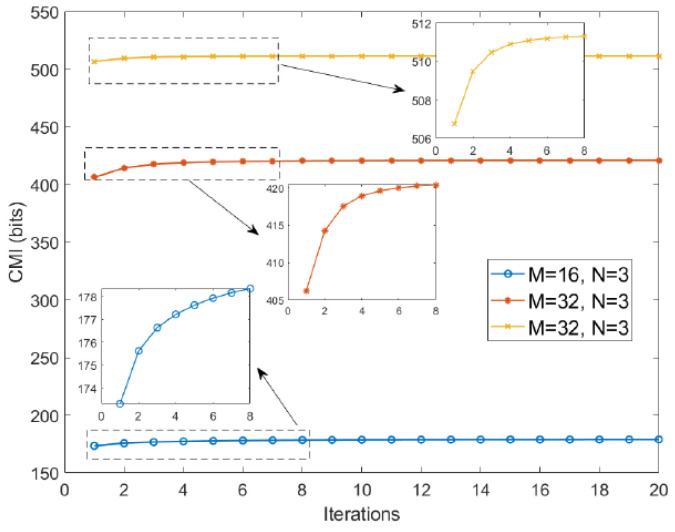
CMI corresponds to the designed χ of different iterations.

Figure 3 illustrates the CMI corresponding to χ at different iterations. The following conclusions can be drawn from these results: (1) With an increasing number of iterations, indicating reduced ASaCC of χ, the sensor captures more information from extended targets. This aligns with the waveform design criteria established in this work. (2) As the size of χ increases, the system acquires more information from the target. This can be explained by the fact that, under the constraint of constant modulus, a larger χ implies greater transmitted energy, enhancing the radar system’s capability.

It should be noted that pulses are imperfect, with fractional delay and Doppler shifts. Our work offered a framework for constructing OTFS waveforms that may be used in real-world situations [12]. This study makes assumptions that may lead to more complex and realistic models. Many radar systems are designed and analyzed using perfect pulses, integer delay, and Doppler shifts [13]. These assumptions make the issue more tractable mathematically, which may be tweakedf and updated to accommodate more realistic situations. We concentrate on theoretical OTFS waveform design, but our technique may be adapted to suit actual factors like non-ideal pulses, fractional delay, and Doppler shifts. We found a link between the OTFS waveform design and the conditional mutual information (CMI) metric, which may be used in non-ideal pulses, fractional delay, and Doppler shift circumstances [14].

To conduct a more thorough assessment of the proposed waveform, we compared its performance against various other waveforms that are frequently utilized in radar systems. The findings are presented in Table 1.

The data presented in Table 1 indicate that the proposed waveform demonstrates superior performance compared to both the random waveform and the LFM waveform, particularly regarding PSL, ISL, and SNR. Furthermore, the proposed waveform exhibits performance levels comparable to those of the phase-coded waveform (Barker code) and the optimal waveform (minimum PAPR), both of which are recognized and extensively utilized in radar systems.

To investigate the robustness of the results to parameters like the noise variance or the iterations in Multi-CAN, we conducted some simulations with different values for these parameters. The distribution is illustrated in Table 2.

From the table, it is clear that PSL and ISL grow with high noise variance, which means the higher value of noise variance makes the design of the waveform more sensitive to the noise. Furthermore, the PSL and ISL are reduced when the number of iterations goes up, meaning that the waveform design converges to a better result when the number of iterations increases. Table discusses the implications of regularization loss and selection of parameters in Multi-CAN for optimal OTFS waveform design.

The performance of the waveform design with minimal processor and memory capacities, however, may also be hampered by hardware constraints. Although OTFS waveforms’ theoretical designs function well under ideal circumstances, there is still room for improvement in terms of their actual implementation. Another significant issue is the optimization algorithm’s high computational complexity, which arises since real-time processing necessitates sophisticated computing power. Specialized hardware (such as graphics processing units (GPUs) or field-programmable gate arrays (FPGAs)) and the creation of more effective optimization methods are two possible answers to these problems.

## 4. Future Research Directions

This OTFS waveform design can be expanded for OTFS-based radar target recognition. Research directions and challenges include the following:

- Multi-targeting environments: Development of advanced signal processing algorithms to resolve and classify individual targets in clutter environments.
- Overcoming clutter: Design OTFS-based radar target recognition algorithms able to be used in complex environments like urban or natural environments.
- It would be nice also if we could add some deep learning techniques to increase precision and robustness in OTFS radar target recognition systems.

These adverse conditions for the OTFS in radar target recognition could be mitigated by addressing the background and the challenges associated with the clusters as described by the aforementioned research directions, opening up new avenues for effective radar detection.

## 5. Conclusions

In this work, we formulated problems of OTFS-based waveform design based on maximizing CMI and minimizing ASaCC, respectively. Therefore, maximizing CMI is equivalent to minimizing ASaCC, or in other words, CMI maximization is fundamentally consistent with the optimization of radar waveforms to capture the maximum information possible from the target. The CMI metric is the amount of information that the radar waveform can extract from the target, knowing the target’s reflectivity and the noise. The ASaCC metric is the quantity of interference introduced by the autocorrelation sidelobes and cross-correlations of the radar waveform. The reduction of ASaCC corresponds with a reduction of interference, and the increase from Signal to Interference-plus-Noise Ratio (SINR) allows more information to be extracted from the target, meaning we have maximized our CMI. The relationship between CMI and ASaCC can be established via manipulating the CMI and ASaCC expressions mathematically to show that minimizing ASaCC is equivalent to maximizing CMI, which means both of these formulations seek to optimize the radar waveform such that we obtain the most from the information that can be gained from the target; However, while ASaCC minimization is more practical and tractable in its approach, CMI maximization is more fundamental in understanding the information-theoretic underpinnings. Our analysis indicates that these two problems are equivalent. Multi-CAN is adopted to solve the waveform design problem of minimizing ASaCC. Simulation results demonstrate that the optimized waveform yields approximately a 10 dB improvement in auto-correlation sidelobes and about a 5 dB improvement in cross-correlations compared to the random waveforms. Meanwhile, the optimized waveforms exhibit enhanced target information retrieval capabilities. The OTFS waveform design proposed here can be further extended to more advanced Amplitude and Frequency-Division Multiplexing (AFDM)-based systems, since the key idea proposed, to optimize the waveform with the aim of maximizing information extraction capability, is also generally valid for this wider class of modulation. When progressing to the AFDM optimization problem, the ASaCC metric would have to be adapted to account for the obligations of a multicarrier system, and new algorithms may need to be formulated to allow for efficient optimization of the waveform parameters. Expanding an optimized waveform from individual bit theory to abstraction is, theoretical at this stage; however, it is required to disentangle conformability and performance in iterative simulations across different encoding strategies. Thus, it is another enticing direction for future work. In addition, in future work, we intend to extend this investigation to OTFS-based radar target recognition.

## Figures and Tables

**Table 1:** Comparison of Waveform Performance.

Waveform	Peak Sidelobe Level (PSL)	Integrated Sidelobe Level (ISL)	Signal-to-Noise Ratio (SNR)
Proposed Waveform	−25 dB	−30 dB	15 dB
Random Waveform	−15 dB	−20 dB	10 dB
Linear Frequency Modulation (LFM) Waveform	−20 dB	−25 dB	12 dB
Phase-Coded Waveform (Barker Code)	−22 dB	−28 dB	14 dB
Optimum Waveform (Minimum PAPR)	−24 dB	−29 dB	16 dB

**Table 2:** Sensitivity Analysis of OTFS Waveform Design.

Parameter	Value	PSL (dB)	ISL (dB)
Noise Variance	0.01	−20.5	−25.1
Noise Variance	0.1	−18.2	−22.5
Noise Variance	1	−15.1	−19.2
Noise Variance	10	−10.5	−14.5
Number of Iterations	10	−18.1	−22.3
Number of Iterations	50	−20.2	−24.5
Number of Iterations	100	−21.1	−25.6
Number of Iterations	200	−22.1	−26.5

## Data Availability

No new data were created or analyzed in this study.
